# Initial in vitro screening approach to investigate the potential health and environmental hazards of Envirox™ – a nanoparticulate cerium oxide diesel fuel additive

**DOI:** 10.1186/1743-8977-4-12

**Published:** 2007-12-05

**Authors:** Barry Park, Patricia Martin, Chris Harris, Robert Guest, Andrew Whittingham, Peter Jenkinson, John Handley

**Affiliations:** 1 Oxonica, 7 Begbroke Science Park, Sandy Lane, Yarnton, Oxfordshire, UK; 2 Product Assessment & Regulatory Compliance, Sydney Road, Cradley Heath, West Midlands, UK; 3 SafePharm Laboratories Ltd, London Road, Shardlow, Derby, UK

## Abstract

Nanotechnology is the new industrial revolution of the 21st Century as the various processes lead to radical improvements in medicine, manufacturing, energy production, land remediation, information technology and many other everyday products and applications. With this revolution however, there are undoubted concerns for health, safety and the environment which arise from the unique nature of materials and processes at the nanometre scale.

The in vitro assays used in the screening strategy are all validated, internationally accepted protocols and provide a useful indication of potential toxicity of a chemical as a result of effects on various toxicological endpoints such as local site of contact (dermal) irritation, general cytotoxicity and mutagenicity.

The initial in vitro screening strategy described in this paper to investigate the potential health implications, if any, which may arise following exposure to one specific application of nanoparticulate cerium oxide used as a diesel fuel borne catalyst, reflects a precautionary approach and the results will inform judgement on how best to proceed to ensure safe use.

## Background

Nanotechnology is the production and application of structures, devices and systems by controlling shape and size at the nanometre scale (1 nanometre = 1 × 10^-6 ^mm). The range of structures covered by the term nanotechnology is also extensive and encompasses existing materials with at least one dimension of < 100 nm and includes nanoparticulates of various metals and their oxides, as well as more diverse engineered nanomaterials such as carbon nanotubes (CNT) and fullerenes.

The downstream applications of nanotechnology are equally diverse, from electronics and engineering, medicinal/medical diagnostic devices, remediation of environmental pollution, personal care products and also food/beverages as well as information technology. The general public is already exposed to a variety of consumer products containing nanoparticulates e.g. titanium dioxide in paints, and cosmetic products, e.g. sunscreens containing zinc oxide and/or titanium dioxide.

Envirox™ is a cerium oxide based fuel borne catalyst and supplied as a dispersion of 2% w/v cerium oxide in a hydrocarbon carrier (which is wholly compatible with diesel). This is diluted with diesel for delivery to the vehicle at a ratio of 1:4000, yielding a final cerium oxide concentration in the vehicle diesel fuel of 5 ppm. The efficacy of Envirox™ is primarily due to the vastly increased surface area of the nanoparticulate cerium oxide. Once in the combustion chamber nanoparticulate cerium oxide works to modify the combustion of diesel fuel while extending and improving fuel burn. In addition the inclusion of nanoparticulate cerium oxide reduces the temperature at which carbon combusts. This feature provides a secondary benefit to the performance of a diesel fuel engine, as it facilitates the removal of hard carbon deposits and soot, which further increases fuel efficiency and potentially reduces wear in the engine and, most importantly, results in reduced particulate emissions from the exhaust.

Despite the fact that humans have been exposed to nano-sized particles throughout their evolution from natural/anthropogenic sources, it is the proliferation of the new (nano)materials which has prompted concerns to be raised [1] over human health and environmental safety following their suspected inevitable release into the environment at some stage of the 'product application' lifecycle. These concerns are intrinsically inter-linked with the extremely small size of these particles. There is an inverse relationship between particle size and surface area, i.e. the smaller the particle the larger the surface area and the greater the proportion of atoms or molecules which are distributed on the surface rather than the interior of the material. These surface changes may lead to an increase in the number of 'reactive sites' on the particle which in turn may lead to an increase in (eco)-toxicological effects [2,3].

In 2003 the UK Government commissioned a review, conducted by the Royal Society and the Royal Academy of Engineering [4], into the likely developments in nanotechnology and an examination of whether this would raise new health and safety, environmental, social or ethical issues which might lead to a requirement for new controlling legislation. This report, together with others [5,6], identified a dearth of eco/toxicological hazard information on nanomaterials that needed to be supplemented prior to better understanding the potential risks, if any, posed by manufactured nanoparticulates.

Recent publications [7-9] have begun the process required to address the development of sound scientific strategies for the safety evaluation, or hazard identification and thence risk assessment of nanomaterials. The above publications agree that any robust approach to hazard identification for nanomaterials may be predicated upon the following three elements:

- physical-chemical characterisation

- *in vitro *assays, and

- *in vivo *studies.

Robust characterisation of each test material is an essential prerequisite of any investigation to ensure that comparison of results between laboratories is based on sound science. This characterisation may include measurement of some, or all, of the following where appropriate: particle size and distribution, shape, agglomeration state, crystal structure, chemical composition, porosity, surface area, surface charge and surface chemistry.

*In vitro *assays of the test material in any given matrix allow the examination of specific biological response(s) and/or mechanism of action under controlled conditions which are not easily studied in complex *in vivo *situations. The *in vitro *assays used in the current preliminary human safety assessment were employed to investigate local toxicological effects (skin irritation), any immediate effects on the structure, function or pathology of cells (cytotoxicological effects) and whether there was any initial adverse effect on the cell's genetic material (Ames test) using a variety of readily available and well-characterised cells in culture. Additional tests were conducted to address the potential for adverse ecotoxicological and environmental fate effects. The results of all these initial tests will be used to inform the stepwise development of a comprehensive testing and safety assessment strategy for nanoparticulate cerium oxide.

The approach outlined in this paper describes an initial *in vitro *preliminary screening strategy to examine the potential for human health hazards following exposure to nanoparticulate cerium oxide. All of the tests conducted have included non-nanoparticulate cerium oxide as a reference material for comparison with nano cerium oxide. The work was carried out within the departments for Alternative Testing and Genetic Toxicology at SafePharm Laboratories Ltd (SPL), Derby UK and is part of a broader investigation of potential health effects.

## Results

### Characterisation of test materials

Data generated from a series of tests to characterise the two forms of cerium oxide used as test materials in the subsequent assessments of toxicological potential are shown in Table 1.

**Table 1:** Physico-chemical characteristics of the test materials

**Test**	**Property**	**Nano**	**Non-Nano**
XRD	Crystal Form	Cerianite	Cerianite
			
EDX	Gross Elemental Analysis	Ce, O	Ce, O
			
BET	Surface Area	94.7 m^2^/g	2.64 m^2^/g
	Mean Particle Size	9 nm	320 nm
			
XPS	Surface Chemistry	Ce, O	Ce, O

**Figure 1:**
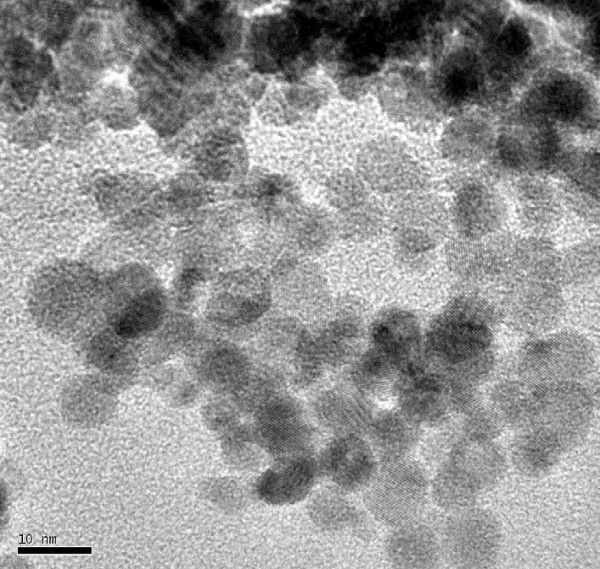
Nanoparticulate cerium oxide.

**Figure 2:**
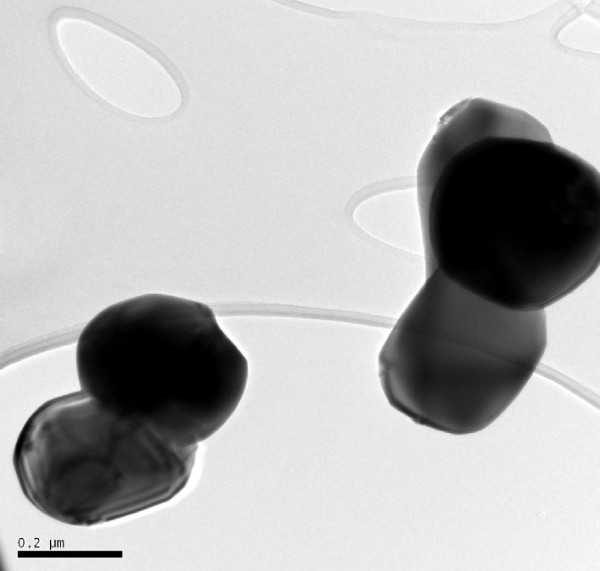
Non-nanoparticulate cerium oxide.

Transmission Electron Microscopy (TEM) photographs of each of the nanoparticulate and non-nanoparticulate cerium oxide samples are shown in Figures 1 and 2. There are significant differences in particle size in the two test material samples and this is clearly illustrated in the photographs.

### Epiderm™ skin model

Table 2 illustrates the mean OD_540 _and mean viability for tissues exposed to the test materials. The nano-cerium oxide and the non-nano cerium oxide test materials did not directly reduce MTT.

**Table 2:** Results of the Epiderm™ skin model study

**Test Materials**	**Exposure time (mins.)**	**Mean OD_540_**	**% Viability**	**ET_50 _mins.**	**MIP**
*NEGATIVE CONTROL*	960	1.685	100		
	1440				
NANO-CeO_2_	960	2.010	119.29	1517.18	<0.01
	1200	1.138	67.54		
	1440	1.089	64.63		
NON-NANO CeO_2_	960	1.994	92.87	> 1440	0.03
	1200	1.975	97.36		
	1440	1.729	85.23		
*POSITIVE CONTROL *1% TX_100_	240	1.035	61.42	260.10	<0.18
	360	0.212	12.58		
	480	0.095	5.64		
*REFERENCE MATERIAL *20% SLS	15	0.983	58.34	20.68	1.00
	30	0.710	42.14		
	60	0.273	16.20		
	120	0.100	5.93		

The ET_50 _values for the nano cerium oxide, 1% (w/v) Triton X-100 the positive control and 1% of 20% (w/v) SLS a standard reference material were 1517.18, 260.10 and 20.68 minutes respectively. The non-nano cerium oxide was assayed at a later time than the nano cerium oxide material and the corresponding ET_50 _values for non-nano cerium oxide, the positive control and the reference material were > 1440, 410.60 and 46.22 minutes respectively.

Using the equation below the Mean Irritation Potential (MIP) for both the nano- and non-nano cerium oxide test materials was calculated and determined as 0.01 for nano cerium oxide and 0.03 for non-nano cerium oxide.

$$\text{MIP calculation equation}:\text{MIP}=\frac{{\text{ET}}_{50}\;(\text{reference material})}{{\text{ET}}_{50}\;(\text{test material})}$$

Since both nano and non-nano cerium oxide test materials had MIP values which were < 0.8 neither was considered to have the potential to be an *in vivo *skin irritant [10].

Based upon a recent statement from the European Centre for the Validation of Alternative Methods (ECVAM) the Epiderm model reliably identifies skin irritants [11] but negative results may require further testing in line with current guidance for this endpoint as described in the OECD TG 404.

### Cytotoxicity assay

The mean cytotoxicity/reactivity grades, related to the concentration and toxicity of soluble components diffusing from the test sample, are illustrated in Table 3. The negative control (polypropylene pipette tips) had a cytotoxicity grade of 0 and the positive control (tin-impregnated PVC strips) showed evidence of a moderate cytotoxicity with a grade 3 score; thus indicating that the assay system is reliable. Both non-nano particulate cerium oxide and nanoparticulate cerium oxide showed no evidence of cytotoxicity and each had a cytotoxicity grade of 0.

**Table 3:** Results of the cytotoxicity testing

**TEST MATERIAL**	**MEAN CYTOTOXICITY/REACTIVITY GRADE**
Culture medium (Control)	0
*Negative control *(polypropylene pipette tips)	0
*Positive control *(tin-impregnated PVC strips)	3
'Non-nano' particulate CeO_2_	0
Nano-particulate CeO_2_	0

### Ames test

Neither of the two cerium oxide particulate samples (non-nano cerium oxide and nano-cerium oxide) were toxic to *Salmonella *strain TA100 when tested in a preliminary toxicity assay and nor did either of the two test materials cause any visible reduction in the growth of the bacterial background lawn at any dose level. Therefore the two cerium oxide particulate test materials were tested up to the maximum recommended dose level of 5000 μg/plate. Non-nano cerium oxide precipitation was not observed on the plates at any of the doses tested in either the presence or absence of S9-mix. A cream-coloured film was observed at 1500 μg/plate and above with an associated precipitateat 5000 μg/plate in the case of the nanoparticulate cerium oxide tested material (the observation of a precipitate of a test sample is a routine means of ensuring that the test has been performed adequately). These observations did not, however, prevent the scoring of revertant colonies and confirmed that the samples were tested up to a maximal dose level.

There were no significant increases in the frequency of revertant colonies recorded for any of the strains of *Salmonella*, at any dose level, either with or without metabolic activation, for either the non-nano cerium oxide or nanoparticulate cerium oxide (Table 4, 5, 6, 7, 8, 9). All of the positive control chemicals used in the study induced marked increases in the frequency of revertant colonies thus confirming the activity of the S9-mix and the sensitivity of the bacterial strains.

**Table 4:** Results of bacterial gene point mutation assay. Cerium oxide – non-nanoparticulate. Summary of mean revertant colonies obtained

**Experiment 1**											
Substance	Dose Level (μg/plate)	TA100		TA1535		TA102		TA98		TA1537	
		
		Mean ± S.D.		Mean ± S.D.		Mean ± S.D.		Mean ± S.D.		Mean ± S.D.	
		
		(-S9)	(+S9)	(-S9)	(+S9)	(-S9)	(+S9)	(-S9)	(+S9)	(-S9)	(+S9)
DMSO	100 μl	83 ± 7	64 ± 5	40 ± 2	12 ± 2	309 ± 24	333 ± 29	21 ± 5	26 ± 4	10 ± 2	17 ± 3
Cerium Oxide Non-nano	50	85 ± 12	73 ± 11	37 ± 5	13 ± 1	333 ± 28	361 ± 29	19 ± 4	29 ± 2	12 ± 2	19 ± 6
	150	96 ± 7	70 ± 9	34 ± 3	11 ± 2	341 ± 21	359 ± 7	19 ± 3	29 ± 3	13 ± 3	18 ± 3
	500	80 ± 12	76 ± 10	35 ± 7	11 ± 2	345 ± 9	367 ± 11	22 ± 7	29 ± 6	13 ± 3	20 ± 4
	1500	69 ± 10	75 ± 11	34 ± 2	13 ± 6	338 ± 10	350 ± 26	16 ± 2	25 ± 3	12 ± 5	21 ± 5
	5000	84 ± 7	73 ± 9	36 ± 3	10 ± 2	313 ± 21	354 ± 14	16 ± 4	31 ± 4	11 ± 1	22 ± 1

**Table 5:** Results of bacterial gene point mutation assay. Cerium oxide – non-nanoparticulate. Summary of mean revertant colonies obtained

**Experiment 2**											
Substance	Dose Level (μg/plate)	TA100		TA1535		TA102		TA98		TA1537	
		
		Mean ± S.D.		Mean ± S.D.		Mean ± S.D.		Mean ± S.D.		Mean ± S.D.	
		
		(-S9)	(+S9)	(-S9)	(+S9)	(-S9)	(+S9)	(-S9)	(+S9)	(-S9)	(+S9)
DMSO	100 μl	93 ± 16	105 ± 13	27 ± 7	9 ± 1	345 ± 17	374 ± 13	20 ± 3	27 ± 7	8 ± 3	13 ± 4
Cerium Oxide Non-nano	50	87 ± 10	115 ± 10	32 ± 2	11 ± 7	349 ± 42	352 ± 31	16 ± 7	23 ± 2	15 ± 4	19 ± 4
	150	95 ± 4	91 ± 8	28 ± 7	14 ± 2	343 ± 20	397 ± 21	20 ± 3	23 ± 2	8 ± 4	19 ± 5
	500	94 ± 13	89 ± 4	37 ± 5	9 ± 1	349 ± 27	364 ± 23	18 ± 2	29 ± 3	9 ± 1	17 ± 3
	1500	82 ± 2	99 ± 2	33 ± 12	9 ± 3	351 ± 22	380 ± 10	17 ± 2	28 ± 4	8 ± 4	21 ± 6
	5000	107 ± 2	97 ± 13	26 ± 4	10 ± 2	350 ± 49	382 ± 21	18 ± 4	23 ± 6	15 ± 5	19 ± 3

S.D. Standard deviation

**Table 6:** Results of bacterial gene point mutation  assay. Cerium oxide - nanoparticulate. Summary of mean revertant  colonies obtained

**Experiment 1**											
		TA100		TA1535		TA102		TA98		TA1537	
		
Substance	Dose Level (μg/plate)	Mean ± S.D.		Mean ± S.D.		Mean ± S.D.		Mean ± S.D.		Mean ± S.D.	
		
		(-S9)	(+S9)	(-S9)	(+S9)	(-S9)	(+S9)	(-S9)	(+S9)	(-S9)	(+S9)
DMSO	100 μl	118 ± 7	97 ± 13	39 ± 3	32 ± 6	347 ± 29	351 ± 4	28 ± 3	36 ± 7	13 ± 5	22 ± 2
Cerium Oxide Nano	50	100 ± 3	109 ± 17	37 ± 2	29 ± 13	356 ± 10	363 ± 25	23 ± 5	36 ± 2	11 ± 5	18 ± 4
	150	88 ± 13	115 ± 9	40 ± 3	36 ± 1	360 ± 13	381 ± 20	25 ± 6	35 ± 6	12 ± 5	15 ± 5
	500	84 ± 16	114 ± 20	33 ± 3	36 ± 3	377 ± 12	350 ± 17	26 ± 6	35 ± 7	14 ± 5	17 ± 4
	1500 (F)	101 ± 13	115 ± 8	28 ± 4	32 ± 9	343 ± 38	330 ± 17	22 ± 3	35 ± 1	14 ± 4	24 ± 2
	5000 (F+M+P)	101 ± 4	113 ± 7	39 ± 3	38 ± 2	343 ± 16	332 ± 14	23 ± 4	37 ± 4	12 ± 2	20 ± 5

**Table 7:** Results of bacterial gene point mutation  assay. Cerium oxide - nanoparticulate. Summary of mean revertant  colonies obtained

**Experiment 2**											
Substance	Dose Level (μg/plate)	TA100		TA1535		TA102		TA98		TA1537	
		
		Mean ± S.D.		Mean ± S.D.		Mean ± S.D.		Mean ± S.D.		Mean ± S.D.	
		
		(-S9)	(+S9)	(-S9)	(+S9)	(-S9)	(+S9)	(-S9)	(+S9)	(-S9)	(+S9)
DMSO	100 μl	97 ± 14	132 ± 8	28 ± 6	30 ± 11	394 ± 9	380 ± 25	21 ± 5	38 ± 7	12 ± 10	15 ± 6
Cerium Oxide Nano	50	99 ± 19	123 ± 17	32 ± 2	29 ± 5	359 ± 28	397 ± 39	21 ± 4	33 ± 10	12 ± 1	13 ± 6
	150	98 ± 20	127 ± 10	35 ± 6	40 ± 6	361 ± 32	391 ± 32	22 ± 4	32 ± 2	14 ± 7	12 ± 4
	500	84 ± 6	126 ± 11	35 ± 11	32 ± 4	382 ± 29	350 ± 7	21 ± 4	32 ± 5	14 ± 4	16 ± 4
	1500 (F)	92 ± 8	133 ± 7	36 ± 8	36 ± 7	361 ± 43	313 ± 29	19 ± 5	37 ± 4	9 ± 3	12 ± 3
	5000 (F+P+M)	95 ± 3	121 ± 22	28 ± 5	29 ± 3	371 ± 24	353 ± 36	19 ± 3	26 ± 3	9 ± 1	17 ± 2

S.D. Standard deviation

F Film

P Precipitate

M Manual Count

**Table 8:** Results of bacterial gene point mutation  assay. Positive control data.

**Experiment 1**										
	TA100		TA1535		TA102		TA98		TA1537	
	
	(-S9)	(+S9)	(-S9)	(+S9)	(-S9)	(+S9)	(-S9)	(+S9)	(-S9)	(+S9)
Compound	ENNG	2AA	ENNG	2AA	MMC	DAN	4NQO	BP	9AA	2AA
Dose Level	3 μg	1 μg	5 μg	2 μg	0.5 μg	10 μg	0.2 μg	5 μg	80 μg	2 μg
Mean ± S.D.	716 ± 35	669 ± 23	731 ± 71	212 ± 4	1338 ± 103	717 ± 38	238 ± 17	213 ± 8	789 ± 79	305 ± 7

**Table 9:** Results of bacterial gene point mutation  assay. Positive control data.

**Experiment 2**										
	TA100		TA1535		TA102		TA98		TA1537	
	
	(-S9)	(+S9)	(-S9)	(+S9)	(-S9)	(+S9)	(-S9)	(+S9)	(-S9)	(+S9)
Compound	ENNG	2AA	ENNG	2AA	MMC	DAN	4NQO	BP	9AA	2AA
Dose Level	3 μg	1 μg	5 μg	2 μg	0.5 μg	10 μg	0.2 μg	5 μg	80 μg	2 μg
Mean ± S.D.	687 ± 88	1055 ± 181	706 ± 38	304 ± 49	1370 ± 86	832 ± 53	247 ± 8	353 ± 84	1909 ± 192	207 ± 44

S.D. Standard deviation

ENNG N-Ethyl-N'-nitro-N-nitrosoguanidine

MMC Mitomycin C

4NQO 4-Nitroquinoline-1-oxide

9AA 9-Aminoacridine

2AA 2-Aminoanthracene

DAN 1,8-dihydroxyanthraquinone

BP Benzo-a-Pyrene

### *Daphnia magna *immobilisation study

There were no toxic effects observed i.e. no immobilisation occurred at any time period during this test.

The 48-hour EC_50 _for nano and non-nano cerium oxide to *Daphnia magna magna *based on nominal test concentrations was greater than 100% (v/v) saturated solution (see Table 10) and correspondingly the No Observed Effect Concentration (NOEC) was 100% (v/v) saturated solution.

**Table 10:** Cumulative immobilisation data in definitive Daphnia test

**Nominal concentration (% v/v saturated solution)**	**Cumulative immobilised Daphnia (initial population: 10 per replicate)**							
	
	**24-hour**				**48-hour**			
	
	**Number per replicate**		**Total**	**%**	**Number per replicate**		**Total**	**%**
**Control:**								
R1	0				0			
R2	0		0	0	0		0	0
R3	0				0			
R4	0				0			

**100% Nano cerium oxide:**								
R1	0				0			
R2	0		0	0	0		0	0
R3	0				0			
R4	0				0			

**100% Non nano cerium oxide:**								
R1	0				0			
R2	0		0	0	0		0	0
R3	0				0			
R4	0				0			

	R_1_	R^2^			R_1_	R_2_		
**Positive control **(mg/l) (potassium dichromate):								
0.32	0	0	0	0	0	0	0	0
0.56	0	0	0	0	0	0	0	0
1.0	2	4	6	30	10	10	20	100

R1 – R4 = Replicates 1 to 4

### Activated sludge respiration inhibition study

The 3-hour EC_50 _for inhibition of respiration of activated sewage sludge bacteria for both nano cerium oxide and non-nano cerium oxide test materials was greater than 1000 mg/l (see Table 11). There was no effect on the respiration of activated sewage sludge following a 3-hour exposure to either of the test materials at 1000 mg/l i.e. No Observed Effect Concentration (NOEC) = 1000 mg/l.

**Table 11:** Oxygen consumption rates and percentage inhibition values in the definitive ASRIT test after 3-h contact time

**Nominal concentration (mg/l)**	**Initial O_2 _reading (mg O_2_/l)**	**Measurement period (minutes)**	**Final O_2 _reading (mg O_2_/l)**	**O_2 _consumption rates (mg O_2_/l/min)**	**% inhibition**
**Control**:					
R1	6.9	10	2.9	0.40	-
R2	6.6	10	2.4	0.42	-
R3	6.9	10	2.8	0.41	-
R4	6.3	8	3.0	0.41	-

**Nano cerium oxide**:					
1000 R1	6.7	10	2.5	0.42	[2]
1000 R2	6.8	10	2.8	0.40	2
1000 R3	6.5	9	2.9	0.40	2

**Non-nano cerium oxide**:					
1000 R1	6.7	9	3.0	0.41	0
1000 R2	6.7	10	2.6	0.41	0
1000 R3	6.8	10	2.8	0.40	2

**3,5-dichlorophenol **(Reference material):					
a) 3.2	7.3	10	4.4	0.29	29
b) 10	7.5	10	5.7	0.18	56
c) 32	8.2	10	7.4	0.08	80
d) 3.2	7.1	10	4.2	0.29	29
e) 10	7.7	10	6.0	0.17	59
f) 32	8.2	10	7.5	0.07	83

R1–R2 controls for nano cerium oxide test

R3–R4 controls for non-nano cerium oxide test

(a – c) reference samples for nano cerium oxide test

(d – f) reference samples for non nano cerium oxide test

## Discussion

Literature data on the potential adverse health effects of particulate cerium oxide are not extensive [12] and an assessment of existing data suggests that the inherent toxicity of cerium oxide is low. The authors are not aware of any reviews of nanoparticulate cerium oxide toxicology appearing in any of the more well-known journals where English is the language of choice. However, it should be remembered that a given mass of nanoparticles has a much greater surface area than the same mass of fine, yet respirable particles (see Table 12 from [13]) and thus is potentially more reactive at the cellular level.

**Table 12:** Correlation of particle diameter with surface area

Airborne mass concentration (μg/m^3^)	Particle diameter (μm)	Particles/ml of air	Particle surface area (μ^2^/ml air) (μ^2^/ml air)
10	2	1.2	24
10	0.5	153	120
10	0.02	2400000	3016

Based upon earlier inhalation studies with a variety of nanoparticles of differing solubility [14] and surface reactivity (but not cerium oxide), effects reported at the cellular level included those associated with the defensive activities of lung macrophages engaged in the phagocytosis of these nanoparticles, and, to the concurrent induction of oxidative stress coupled to (pro)-inflammatory processes [15].

A pivotal feature of the preliminary safety studies reported above was to mirror the approach already routinely applied by industry and regulators when assessing health and environmental hazards of chemicals within Europe. Cerium oxide is an 'existing chemical' (listed on the European Inventory of Existing Commercial Chemical Substances: EINECS), only the form i.e. the size of the chemical particles has been modified in the nano cerium oxide test material. The objective of the preliminary screening strategy was to compare the potential toxicity of nano and non-nano forms of cerium oxide in a limited set of in vitro assays. The results would then inform judgement on the strategy to be adopted for a comprehensive safety assessment should these data indicate that the nano cerium oxide posed any additional health risks compared to non-nano cerium oxide.

Results of the preliminary in vitro screening approach reported here with a limited number of assays are clearly negative. Based on the available hazard data these studies do not raise any significant concerns for potential adverse human health or environmental effects as a result of limited localised exposure of cells or aquatic organisms to nano cerium oxide. Indeed the current information from these preliminary studies does not indicate any difference in cellular response following exposure to nanoparticulate cerium oxide when compared to that of non nano cerium oxide.

The preliminary safety testing approach described in this paper is consistent with the initial part of a screening test battery approach to identify whether or not the nanoparticulate form of a substance is likely (or not) to cause significant different adverse effects to those of the non-nano substance which emerged from an opinion issued by the EU Scientific Committee on Emerging and Newly Identified Health Risks (SCENIHR) on the appropriateness of existing safety testing approaches for the assessment of any potential risks associated with nanotechnologies [16].

The SCENIHR report states that where the nanoparticulate has similar hazard properties to other physical forms of the substance then existing studies (*in vitro*) combined with information on the physicochemical characteristics may lead to the overall conclusion that further work on hazard assessment of the nanoparticulate form may not be necessary. However, we recognise that the *in vitro *hazard data currently reported is limited, it is simply Phase 1 of a comprehensive safety assessment work programme, and that further work is needed before we can be satisfied of the safety in use of nano cerium oxide in Envirox™ as a diesel fuel additive.

Further support for the stepwise approach to safety assessment of nano cerium oxide reported here is provided by the UK Committees on Toxicity, Mutagenicity and Carcinogenicity of Chemicals in Food, Consumer Products and the Environment (COT, COM, COC) [17]. These august bodies were tasked to provide advice on the development of nanotechnology to the UK Government and proposed a systematic tiered approach for initial toxicological studies on novel nanomaterials based on in vitro screening of selected materials to be supported by biodistribution studies to aid in the identification of the cell types for study, followed by appropriate in vivo testing to address any endpoints of concern.

## Conclusion

The preliminary screening studies reported in this paper employed an *in vitro *approach to hazard identification of nanoparticulate cerium oxide and benchmark any effects by comparison with non-nano cerium oxide.

The initial screening studies for human health which included local site of contact (dermal) irritation, general cytotoxicity and mutagenicity and environmental effects (acute *Daphnia magna *toxicity and activated sludge respiration inhibition) demonstrated no differences in biological effects potential between nano and non-nano cerium oxide.

These data support the conclusion of a lack of any additional (eco)-toxicological effects potential for nano-cerium oxide when compared to that of non-nano cerium oxide in a number of in vitro assays undertaken as a preliminary toxicity screen.

## Methods

### Characterisation of the test materials used in subsequent *in vitro *assays

Cerium oxide samples used in these assays were characterised using a variety of analytical techniques including XRD (X-ray diffraction), EDX (Energy Dispersive X-ray analysis), BET (Brunauer Emmett Teller Surface Area), XPS (X-ray Photoelectron Spectroscopy) and TEM (Transmission Electron Microscopy).

Sample (1) – Nanoparticulate cerium oxide was received as an aqueous slurry at an approximate concentration of 8%w/w. Physico-chemical characterisation was conducted on this form (as supplied) or after freeze drying.

Sample (2) – Non-nanoparticulate cerium oxide was defined as "Cerium (IV) oxide, powder, <5 micron, 99.9% pure, Aldrich catalogue number 211575".

### *In vitro *assays

Three *in vitro *assay protocols were used in this screening approach to investigate potential health hazards following exposure to nanoparticulate and non-nanoparticulate cerium oxide and comprised:

(a) EpiDerm™ Skin Irritation Test

(b) BS EN ISO 10993-5 Cytotoxicity assay

(c) 'Ames test' – OECD 471/EC B14 method

#### (a) The EpiDerm™ 'EPI-200' human epidermal model

The EpiDerm™ EPI-200 human epidermal model from MatTek Corporation (Ashland, MA, USA) was utilised in this preliminary screening approach for assessment of the safety of Envirox™ since the skin is a potential route of exposure during the formulation and use of the nano-cerium oxide containing diesel fuel additive when accidental skin contamination might reasonably be expected to occur.

The EpiDerm™ study methodology used in this investigation was developed in-house at MatTek [18]. An exposure time range-finding study was performed to establish the appropriate range of exposure times to be used in the main study.

#### (b) BS EN ISO 10993-5 Cytotoxicity Test

The BS EN ISO 10993-5 cytotoxicity test was selected as the endpoint of the assay is a qualitative and semi-quantitative estimate of toxicity that is non-specific in terms of mechanism. Therefore, it is a robust procedure for the detection of any potential for cell toxicity and may be considered to be a highly sensitive *in vitro *indicator test for the prediction of *in vivo *toxicity.

The assay was used as described in the ISO methodology [19] and a comparison made between the responses of test materials and those of the positive control (tin-impregnated plastic strip), the negative control (polypropylene tip) and culture medium control to determine if there were any differences in morphology or cell numbers. This qualitative evaluation was translated into a semi-quantitative score as 'cytotoxic response' rated on a scale of 0 to 4, as shown in Table 13.

**Table 13:** Semiquantitative score of cytotoxic response for BS EN ISO 10993-5 Cytotoxicity Test

**Grade**	**Cytotoxicity/Reactivity**	**Description of Cytotoxicity/Reactivity Zone**
0	None	No detectable zone around or under specimen
1	Slight	Some malformed or degenerated cells
2	Mild	Zone limited to area under specimen
3	Moderate	Zone extends 0.5 to 1.0 cm beyond specimen
4	Severe	Zone extends greater than 1.0 cm beyond specimen but does not involve entire dish

#### (c) Ames test

The OECD 471 Ames test was selected as the logical first assay to determine the mutagenic potential of nano and non-nano cerium oxide because it has both high sensitivity and specificity. The bacterial gene point mutation assay is the primary *in vitro *screening method used in the evaluation of chemicals, pharmaceuticals, plant protection products and other materials. It has been comprehensively validated over the last 30 years and has been applied in the testing of tens of thousands of products.

*Salmonella typhimurium *strains TA 98, 100, 102, 1535 & 1537 were used as recommended in the OECD 471 test guideline methodology (see Table 14) for the general screening of chemicals for mutagenicity [20] and results confirmed by statistical methods [21].

**Table 14:** Chemical effects on various Salmonella typhimurium strains

TA100	sensitive to agents inducing base-pair substitution
TA1535	sensitive to agents inducing base-pair substitution
TA102	sensitive to agents inducing base-pair substitution
TA1537	sensitive to agents inducing frame-shift mutations
TA98	sensitive to agents inducing frame-shift mutations

A range of concentrations (50 – 5000 μg/plate in triplicate based on a preliminary test) of both nano and non-nano cerium oxide were prepared as suspensions in dimethyl sulphoxide using a vortex mixer and sonication since they were not soluble in water or common solvents. A solvent control and a range of positive control test materials were employed in the assay and included:-

(a) in the absence of metabolic activation (-S9): N-ethyl-N'-nitro-N-nitrosoguanidine (ENNG) at 3 μg/plate for TA100 and 5 μg/plate for TA1535, 9-Aminoacridine (9AA) at 80 μg/plate for TA1537, Mitomycin C (MMC) at 0.5 μg/plate for TA102 and 4-Nitroquinoline-1-oxide (4NQO) at 0.2 μg/plate for TA98, and

(b) in the presence of metabolic activation (+S9): 2-Aminoanthracene (2AA) at 1 μg/plate for TA100, 2 μg/plate for TA1535 and TA1537, Benzo(a)pyrene (BP) at 5 μg/plate for TA98 and 1,8-Dihydroxyanthraquinone (DAN) at 10 μg/plate for TA102.

### Ecotoxicity/environmental fate studies

#### Acute toxicity for *Daphnia magna*

The method was that updated by the OECD Expert Group on Ecotoxicology in April 2004 [22]. As a consequence of the poor water solubility of both nano and non-nano cerium oxide a modification of the standard preparation of aqueous media, as endorsed by ECETOC and OECD [23,24] was employed. This adaptation allowed for the exposure of *Daphnia magna *to a saturated solution of these high purity but poorly water soluble cerium oxide test materials.

#### Activated sludge respiration inhibition test

The methodology is described in OECD test guideline 209 [16].

The test materials, both nano and non-nano cerium oxide were dispersed directly in water. This approach allowed for assessment of any effects of excess undissolved test material on the activated sludge micro-organisms exoenzymes and also allowed for an evaluation of the uptake of undissolved test material by processes such as phagocytosis which may adversely affect activated sewage sludge. Additionally this procedure allowed for an examination of the entire waste water treatment facility with regards to possible initial contact toxicity.

## Competing interests

The author(s) declare that they have no competing interests.

## Authors' contributions

BP and PM conceived the study, participated in its design and coordination and drafted the manuscript.

CH undertook the nanoparticle characterisation work

RG and AW undertook the Epiderm studies

PJ undertook the cytotoxicity and mutagenicity studies

JH undertook the ecotoxicity studies

All authors read and approved the final manuscript.
